# An rs4705342 T>C polymorphism in the promoter of miR-143/145 is associated with a decreased risk of ischemic stroke

**DOI:** 10.1038/srep34620

**Published:** 2016-10-06

**Authors:** Ye-Sheng Wei, Yang Xiang, Pin-Hu Liao, Jun-Li Wang, You-Fan Peng

**Affiliations:** 1 Department of Clinical Laboratory, the Affiliated Hospital of Youjiang Medical University for Nationalities, Baise 533000, Guangxi, China; 2 Department of Medicine, the Affiliated Hospital of Youjiang Medical University for Nationalities, Baise, 533000, Guangxi, China

## Abstract

The expression of miR-143/miR-145 was up-regulated in ischemic stroke (IS), which may be used as biomarkers and/or therapeutic targets for IS. We aimed to investigate the association of rs4705342 and rs4705343 polymorphisms in the promoter of miR-143/145 with risk of IS. The study population comprised 445 patients with IS and 518 controls. The rs4705342 genotype was analyzed by using a TaqMan Assay and the rs4705343 genotype was determined by using a polymerase chain reaction-restriction fragment length polymorphism assay. Relative expression of miR-143/miR-145 was measured by quantitative real-time PCR. We found that the rs4705342 was associated with a decreased risk of IS (TC vs. TT: adjusted OR = 0.74, 95% CI, 0.57–0.97; CC vs. TT: adjusted OR = 0.53, 95% CI, 0.34–0.83). Haplotype analysis showed that the TC haplotype was associated with an increased risk of IS risk (OR = 1.33, 95% CI, 1.01–1.75), whereas the CT haplotype was associated with a decreased risk of IS risk (OR = 0.68, 95% CI, 0.50–0.92). Importantly, patients carrying the rs4705342TC/CC genotypes had a lower level of miR-145 (*P *= 0.03). We found for the first time that the rs4705342 CC was a protective factor for IS, probably by reducing the level of miR-145.

Atherosclerosis is a chronic inflammatory response of accumulation of white blood cells and proliferation of smooth muscle cell (SMC)^1^. The pathology of atherosclerosis is complicated, but generally, forms atherosclerotic plaques. The obstruction of heart vessels results in coronary artery disease, and the obstruction of brain vessels results in stroke^2^,^3^. In China, stroke is a major cause of death and adult disability, with 2.6 billion new cases and 1.6 billion deaths each year^4^. Ischemic stroke (IS) is the most common type of stroke, accountable for 43–79% of all strokes^4^. To date, several non-genetic risk factors have been identified to contribute to the pathogenesis of IS, such as smoking, hypertension, diabetes and heart diseases^5^,^6^. Reports involving family and twins studies showed that genetic factors may play key roles in the development of IS^7^,^8^,^9^.

MicroRNAs (miRNAs) are small non-coding single-stranded RNAs, which regulate gene expression and play important roles in various biological functions, such as inflammation, atherosclerosis, and stroke^10^,^11^,^12^,^13^,^14^,^15^,^16^. miR-143 and miR-145, located on human chromosome 5, are modulators of SMC phenotype. The expression of miR-143 and miR-145 was up-regulated in both atherosclerosis and IS^11^,^17^,^18^,^19^. Antagomir-mediated prevention of miR-145 level has been found to be atheroprotective^13^,^16^. These findings indicate that miR-145 may be used as a biomarker or therapeutic target for IS^14^.

Previous work has shown that single nucleotide polymorphisms (SNPs) in the promoters of miRNAs may modulate individual’s susceptibility to a variety of human diseases^20^,^21^,^22^,^23^,^24^,^25^,^26^,^27^,^28^,^29^,^30^. Recently, several SNPs in the promoter of miR-143/145 cluster have been identified^21^. Among them, SNPs of rs4705342 T>C and rs4705343 T>C were functional, with the rs4705342T allele having a higher protein-binding affinity and lower promoter activity and the rs4705343C allele displaying a reduced transcriptional activity^29^,^30^. Based on this background, we hypothesized that the 2 SNPs may be related to the risk of IS. To test this hypothesis, we performed a case-control study to examine the association of the rs4705342 T>C and rs4705343 T>C and IS risk in a Chinese population. Moreover, expression levels of miR-143/145 in IS patients and their relationship with the 2 SNPs were also explored.

## Materials and Methods

### Study population

The study protocol was approved by the Ethical Committee of the Affiliated Hospital of Youjiang Medical University for Nationalities. The experiments were performed in accordance with relevant guidelines and regulations. The study population included 518 controls and 445 patients with IS from the department of neurology, Affiliated Hospital of Youjiang Medical University for Nationalities, Guangxi, China between October 2010 and December 2014. Detailed information of inclusion criteria was described in our previous work^31^. Briefly, an established diagnosis of IS was determined according to clinical manifestations and cranial magnetic resonance imaging and/or computed tomography scans. Patients with hemorrhagic, autoimmune or chronic inflammatory diseases were excluded. There were 308 men and 137 women with a mean age (± standard deviation, SD) of 60.1 (±11.0) years in the IS group. Control subjects were healthy volunteers who were selected from physical examination center of the hospital during the same period. The controls were frequency matched to cases on the basis of age and gender. Clinical information, such as hypertension, diabetes, fasting serum levels of total cholesterol (TCH), triglyceride (TG), high-density lipoprotein cholesterol (HDL-C), and low-density lipoprotein cholesterol (LDL-C) was abstracted from medical record review. All participants were unrelated Han Chinese who were consecutively selected from the same geographic region. Informed consent was obtained from all subjects.

### DNA isolation and genotyping

Genomic DNA was extracted from eathylene diamine tetraacetic acid-anticoagulated peripheral blood by using a salting-out method^32^. Genotyping methods were described in detail previously^29^,^30^. Briefly, the rs4705342 T>C genotype was analyzed by using a TaqMan Assay on an ABI 7900HT real-time PCR System (Applied Biosystems, CA, USA), and the rs4705343 T>C genotype was identified by using a polymerase chain reaction-restriction fragment length polymorphism (PCR-RFLP) assay. Two independent research assistants read the results with a blindness of cases and controls. Distilled water was used as a negative control. Ambiguous genotyping results were verified by sequencing analysis.

### Quantitative PCR of miRNA-143 and miR-145

Total RNA was isolated from 1ml of plasma of 46 patients and 46 normal controls using a commercial kit (Qiagen, Hilden, Germany) following the manufacturer’s protocol. One microgram of total RNA was reverse transcribed into cDNA utilizing reverse transcription kits from Ribobio Corp., Guangzhou, China (ssD809230154 for miR-143 and ssD809230156 for miR-145). After cDNA conversion, quantitative PCR was done using Power SYBR Master Mix and ABI 7900HT real-time PCR machine (Applied Biosystems, CA, USA). The primers were purchased from Ribobio Corp., China (ssD809230846 for miR-143 and ssD809230848 for miR-145). U6 was used as an internal control. Relative expression levels of miR-143 and miR-145 were computed using comparative Ct method (2^−ΔCt^).

### Statistical analysis

Statistical analyses were done by using the SPSS 19.0 statistical software package (SPSS Inc., Chicago, IL, USA). Continuous variables were displayed as mean ± SD. If the data were normally distributed, the Student’s *t*-test was used; otherwise, Mann-Whitney U test was used. Categorical variables were expressed as proportions and compared by using chi-squared test. Hardy–Weinberg equilibrium (HWE) was tested by chi-squared test. The association of the rs4705342 T>C and rs4705343 T>C polymorphisms and risk of IS was evaluated by odds ratios (ORs) with 95% confidence interval (CIs). Association analysis and haplotype analysis were performed using SNPstats^33^. ORs were adjusted based on age, gender, hypertension, diabetes mellitus, TCH, TG, HDL-C, and LDL-C using inverse proportional weights analysis. Both average treatment effect (ATE) and average treatment effect on the treated (ATT) were used to estimate adjusted ORs. The statistical significant criteria was considered as *P* < 0.05.

## Results

The clinical characteristics of cases and controls are shown in Table 1. There was no significant difference between the two groups based on age, gender, and HDL-C (*P* > 0.05). The frequencies of hypertension and diabetes mellitus in the IS group were significantly higher than those in the control group (*P* < 0.05). Increased levels of TCH, TG, and LDL-C were observed in the cases compared with the controls (*P* < 0.001).

The association of the rs4705342 T>C and rs4705343 T>C polymorphisms and risk of IS is presented in Table 2. The genotype distributions of the 2 polymorphisms in both cases and controls were in HWE, with *P* values of 0.56 and 0.95 for the rs4705342 T>C and 0.43 and 0.14 for the rs4705343 T>C. Compared with the rs4705342TT genotype, the rs4705342TC and CC genotypes were associated with decreased risks of IS (TC vs. TT: adjusted OR = 0.74, 95% CI, 0.57–0.97, *P* = 0.03; CC vs. TT: adjusted OR = 0.53, 95% CI, 0.34–0.83, *P* *=* 0.006). However, no significant association of the rs4705343 T>C with IS risk was found (Table 2). After stratification analysis, we also failed to find any association between the two polymorphisms and clinical characteristics of IS (Supplemental Tables I and II).

Haplotype analysis of the rs4705342 T>C and rs4705343 T>C polymorphisms was performed. We found that the 2 polymorphisms were in linkage disequilibrium (LD) (D’ = 0.87, r = 0.34). As shown in Table 3, the TC haplotype was associated with an increased risk of IS risk (OR = 1.33, 95% CI, 1.01–1.75, *P* = 0.04), whereas the CT haplotype was associated with a decreased risk of IS risk (OR = 0.68, 95% CI, 0.50–0.92, *P* = 0.012).

**Figure 1:**
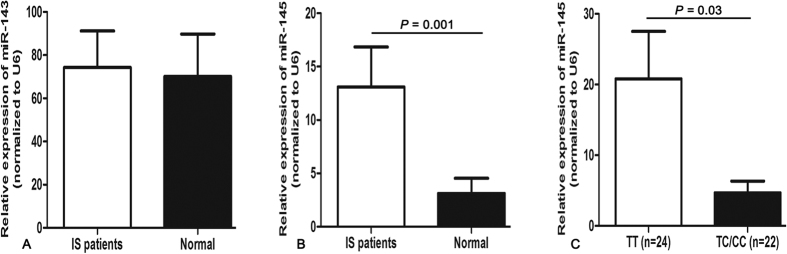
Relative expression of miR-143 and miR-145 in ischemic stroke (IS) patients (n = 46) and normal controls (n = 46). (**A**) No significant difference of miR-143 between IS patients and normal controls. (**B**) Increased level of miR-145 in IS patients compared with normal controls (*P* = 0.001). (**C**) Decreased level of miR-145 in IS patients carrying the rs4705342TC/CC compared with those carrying the rs4705342TT (*P* = 0.03). Data are presented as mean ± standard error.

Additionally, plasma levels of miR-143 and miR-145 were examined among cases and controls. Elevated level of miR-145 but not miR-143 was detected in IS patients. Notably, after correlation analysis of the rs4705342 T>C polymorphism with miR-145 expression, we found that patients carrying the rs4705342TC/CC had a lower level of miR-145 compared with those carrying the rs4705342TT (*P* = 0.03) (Fig. 1). Nevertheless, after comparing the rs4705342 and rs4705343 polymorphisms with relative expression of miR-143 and miR-145 in different gender, we did not find any significant difference (Supplemental Tables III and IV).

## Discussion

This is the first study to evaluate the association between the rs4705342 T>C and rs4705343 T>C polymorphisms in the promoter of miR-143/145 and risk of IS. We demonstrated that the rs4705342 T>C was associated with a reduced risk of IS. The reduced risk was also observed in haplotype analysis. Notably, the rs4705342 T>C was related to abnormal miR-145 expression level in overall analysis but not stratification analysis according to gender. These findings indicate that the rs4705342 T>C may be a protective factor for the etiology of IS.

Atherosclerosis is a major cause of stroke. miR-143 and miR-145 are two SMC-enriched miRNAs, which play important roles in the pathogenesis of atherosclerotic vascular diseases^34^,^35^. Increased levels of miR-143 and miR-145 can reduce proliferation of SMCs^34^,^35^,^36^,^37^. Loss of miR-143 and miR-145 can attenuate the progression of atherosclerosis^38^. Moreover, circulating miR-145 was significantly increased in IS patients and miR-145 was positively correlated to plasma high-sensitivity C-reactive protein^10^,^11^. Taken together, these findings denote that miR-143/145 may be involved in the pathological process of IS. However, not all subjects with altered expression of miR-143/145 develop IS, suggesting that genetic variants may be related to risk of IS.

In 2013, several SNPs in the promoter of miR-143/145 were discovered^21^. Subsequent reports demonstrated that two of them (i.e., rs4705342 T>C and rs4705343 T>C) were functional with different transcriptional activity^29^,^30^. The rs4705343 TC genotype was associated with an increased risk of cervical squamous cell carcinoma^30^, whereas the rs4705342C allele was associated with a decreased risk of essential hypertension^20^ and prostate cancer^29^. In this study, reduced risks of the rs4705342 TC and CC genotypes with IS risk were found. Additionally, the rs4705342C-rs4705343T haplotype had a 0.68-fold decreased risk to develop IS. Previous genome-wide association studies have identified chromosome 5 is a susceptibility loci for IS^39^,^40^,^41^,^42^. Several coding genes in this region have been reported to link to the risk of IS, such as *phosphodiesterase 4D, msh homeobox 2, transforming growth factor beta*, and *glyceraldehyde-3-phosphate dehydrogenase pseudogene 71*^40^,^42^. We hypothesized that non-coding genes in this region may be related to the risk of IS. MiR-143/145 located on 5q32 in human. In the current study, we selected the rs4705342 and rs4705343 polymorphisms in the promoter region of miR-143/145 and got the positive results using both ATE and ATT. Base on the above-mentioned observations, the positive results may be biologically reasonable. Further replications are needed to confirm these findings.

With regard to the potential mechanism, we speculated that the rs4705342 T>C may influence the expression of miR-143 or/and miR-145, and eventually result in the protective effect. We then detected the levels of miR-143 and miR-145 and assessed the expression with the rs4705342 T>C polymorphism. The results confirmed our hypothesis. We found that the rs4705342TC/CC genotypes corresponded to a lower level of miR-145, supporting the idea that genetic polymorphism in the promoter of miRNA may influence the expression of mature miRNA, and finally affect individual’s susceptibility to human diseases. Although it is unknown why only miR-145 was differentially expressed in relation to rs4705342 genotypes, one possible explanation might reside in plasma we used to investigate the expression of miR-143/145. Further studies examining the levels of miR-143/145 in tissue samples may justify these results. Moreover, cell-dependent mechanisms may clarify the reason for this different association.

Although our results are promising, some limitations of the study should be discussed. Firstly, relatively small sample size may result in insufficient power to detect the association of the rs4705342 T>C and rs4705343 T>C with IS risk. Further validations are necessary in independent studies. Secondly, this study is based on hospitalized controls, which may lead to a selection bias. Population-based replication studies are valuable to confirm these findings. Finally, genotype-phenotype analysis cannot be done due to lack of data on smoking and alcohol consumption.

In conclusion, this study provides evidence that the rs4705342 T>C in the promoter of miR-143/145 was associated with a decreased risk of IS, probably by reducing the level of miR-145. These findings, if validated in large cohort studies, may help us to understand the precise effect of miRNA-related SNPs on the etiology of IS.

## Additional Information

**How to cite this article**: Wei, Y.-S. *et al*. An rs4705342 T>C polymorphism in the promoter of miR-143/145 is associated with a decreased risk of ischemic stroke. *Sci. Rep.* **6**, 34620; doi: 10.1038/srep34620 (2016).

## Supplementary Material

Supplementary Information

## Figures and Tables

**Table 1:** Clinical characteristics of the study population.

Variables	Controls (n = 518)	IS (n = 445)	*P* value
Age (years, mean ± SD)	58.8 (±11.5)	60.1 (±11.0)	0.09
Gender (M/F)	342/176	308/137	0.29
Hypertension, n (%)	104 (20.1)	260 (58.4)	<0.001
Diabetes mellitus, n (%)	48 (9.3)	69 (15.5)	0.003
TCH, mmol/L	4.69 ± 0.75	5.03 ± 0.73	<0.001
TG, mmol/L	1.10 ± 0.34	1.91 ± 1.12	<0.001
HDL-C, mmol/L	1.58 ± 0.35	1.56 ± 0.36	0.53
LDL-C, mmol/L	2.28 ± 0.98	2.66 ± 0.99	<0.001

IS, ischemic stroke; SD, standard deviation; TCH, total cholesterol; TG, triglyceride; HDL-C, high-density lipoprotein cholesterol; LDL-C, low-density lipoprotein cholesterol.

**Table 2:** Associaiton between the rs4705342 T>C and rs4705343 T>C polymorphisms and risk of ischemic stroke.

Polymorphisms	Controls, n = 518 (%)	IS, n = 445 (%)	ATE	*P* value^†^	ATT	*P* value^†^
			Adjusted OR (95% CI)^†^		Adjusted OR (95% CI)^†^	
rs4705342 T>C						
TT	228 (44.0)	234 (52.6)	1.00		1.00	
TC	232 (44.8)	174 (39.1)	0.74 (0.57–0.97)	0.03	0.71 (0.49–0.92)	0.01
CC	58 (11.2)	37 (8.3)	0.53 (0.34–0.83)	0.006	0.51 (0.33–0.81)	0.004
rs4705343 T>C						
TT	230 (44.4)	188 (42.2)	1.00		1.00	
TC	243 (46.9)	209 (47.0)	1.05 (0.81–1.38)	0.71	1.01 (0.74–1.26)	0.67
CC	45 (8.7)	48 (10.8)	0.93 (0.58–1.49)	0.75	0.71 (0.43–1.16)	0.17

IS, ischemic stroke; ATE, average treatment effect; ATT, average treatment effect on the treated; OR, odds ratio; CI, confidence interval.

^†^Adjusted by age, gender, hypertension, diabetes mellitus, TCH, TG, HDL-C, and LDL-C.

**Table 3:** Haplotype analysis of the rs4705342 T>C and rs4705343 T>C polymorphisms with risk of ischemic stroke.

Haplotypes	Controls (%)	IS (%)	OR (95% CI)	*P* value
TT	572 (55.2)	506 (56.9)	1.00	
CC	218 (21.0)	170 (19.1)	0.88 (0.70–1.11)	0.29
TC	116 (11.2)	136 (15.3)	1.33 (1.01–1.75)	0.04
CT	130 (12.5)	78 (8.8)	0.68 (0.50–0.92)	0.012

IS, ischemic stroke; OR, odds ratio; CI, confidence interval.
