# Supplementary material for: An rs4705342 T>C polymorphism in the promoter of miR-143/145 is associated with a decreased risk of ischemic stroke
Source: Sci Rep. 2016 Oct 6;6:34620. doi: 10.1038/srep34620 (PMC5052611; doi:10.1038/srep34620)
Supplement: Supplementary Information [file srep34620-s1.pdf]

An rs4705342 T>C polymorphism in the promoter of miR-143/145 is associated with a decreased risk of ischemic stroke

Ye-Sheng Wei [1]*, Yang Xiang [1], Pin-Hu Liao [2], Jun-Li Wang [1], You-Fan Peng [1]

1.Department of Clinical Laboratory, The Affiliated Hospital of Youjiang Medical University for Nationalities, Baise 533000, Guangxi, China.

2. Department of Medicine, The Affiliated Hospital of Youjiang Medical University for Nationalities, Baise, 533000, Guangxi, China.

Supplemental Table I Clinical variables of ischemic stroke patients with rs4705342 T>C polymorphism.

Variables	rs4705342 T>C		*P* value
	TT (n = 234)	TC/CC (n = 211)	
TCH (mean ± SD)	5.02 ± 0.71	5.03 ± 0.75	0.90
TG (mean ± SD)	1.90 ± 1.12	1.91 ± 1.12	0.97
HDL-C (mean ± SD)	1.54 ± 0.36	1.58 ± 0.36	0.24
LDL-C (mean ± SD)	2.71 ± 0.95	2.61 ± 1.02	0.30

SD, standard deviation; M, male; F, female; TCH, total cholesterol; TG: triglyceride; HDL-C, high-density lipoprotein cholesterol; LDL-C, low-density lipoprotein cholesterol

Supplemental Table II Clinical variables of ischemic stroke patients with rs4705343 T>C polymorphism.

Variables	rs4705343 T>C		*P* value
	TT (n = 188)	TC/CC (n = 257)	
TCH (mean ± SD)	5.04 ± 0.72	5.02 ± 0.73	0.86
TG (mean ± SD)	1.91 ± 1.08	1.91 ± 1.15	0.98
HDL-C (mean ± SD)	1.57 ± 0.38	1.56 ± 0.36	0.77
LDL-C (mean ± SD)	2.62 ± 1.00	2.69 ± 0.98	0.47

SD, standard deviation; M, male; F, female; TCH, total cholesterol; TG: triglyceride; HDL-C, high-density lipoprotein cholesterol; LDL-C, low-density lipoprotein cholesterol

Supplemental Table III. Association of rs4705342 and rs4705343 polymorphisms with expression of miR-143 in different gender

	rs4705342			rs4705343		
	TT	TC/CC	*P* value	TT	TC/CC	*P* value
IS						
Male (n = 31)	89.6 ± 27.6	62.8 ± 39.6	0.21	62.2 ± 60.8	57.9 ± 25.3	0.30
Female (n = 15)	59.9 ± 29.7	69.7 ± 33.6	0.86	127.7 ± 36.3	73.9 ± 24.1	0.54
Controls						
Male (n = 29)	74.6 ± 28.4	62.1 ± 31.3	0.44	54.2 ± 44.7	81.8 ± 50.8	0.11
Female (n = 17)	47.1 ± 44.0	88.1 ± 68.5	0.22	74.4 ± 36.6	65.4 ± 34.7	0.61

IS, ischemic stroke

Relative expression of miR-143 was calculated using comparative Ct method after normalized by U6. Data are presented as mean ± standard error.

Supplemental Table IV. Association of rs4705342 and rs4705343 polymorphisms with expression of miR-145 in different gender

	rs4705342			rs4705343		
	TT	TC/CC	*P* value	TT	TC/CC	*P* value
IS						
Male (n = 31)	10.4 ± 4.2	15.2 ± 10.9	0.23	9.0 ± 6.6	16.9 ± 12.4	0.65
Female (n = 15)	13.6 ± 6.1	15.0 ± 7.2	0.46	11.2 ± 3.9	13.0 ± 3.9	0.89
Controls						
Male (n = 29)	2.9 ± 1.0	5.3 ± 4.8	0.08	5.7 ± 7.3	2.3 ± 1.2	0.07
Female (n = 17)	2.5 ± 1.9	2.8 ± 1.5	0.60	1.6 ± 0.5	3.2 ± 1.4	0.89

IS, ischemic stroke

Relative expression of miR-145 was calculated using comparative Ct method after normalized by U6. Data are presented as mean ± standard error.
